# Chidamide stacked in magnetic polypyrrole nano-composites counter thermotolerance and metastasis for visualized cancer photothermal therapy

**DOI:** 10.1080/10717544.2022.2068697

**Published:** 2022-04-27

**Authors:** Sizhen Wang, Zhiqiang Ma, Zhang Shi, Ying Huang, Tianheng Chen, Lei Hou, Tao Jiang, Feng Yang

**Affiliations:** a Department of Inorganic Chemistry, Pharmacy School, Naval Medical University School, Shanghai, PR China; b College of Pharmacy, Fujian University of Traditional Chinese Medicine, Fujian, PR China; c Department of Radiology, Zhongshan Hospital, Fudan University, Shanghai, PR China; d Department of Nuclear Medicine, Shanghai Fourth People’s Hospital, School of Medicine, Tongji University, Shanghai, PR China

**Keywords:** Magnetic targeting nanocomplex, photothermal therapy, synergistic treatment, dual-modal imaging, polypyrrole

## Abstract

Photothermal therapy (PTT) has become one of the most promising therapies in cancer treatment as its noninvasiveness, high selectivity, and favorable compliance in clinic. However, tumor thermotolerance and distal metastasis reduce its efficacy, becoming the bottleneck of applying PTT in clinic. In this study, a chidamide-loaded magnetic polypyrrole nanocomposite (CMPP) has been fabricated as a visualized cancer photothermal agent (PTA) to counter tumor thermotolerance and metastasis. The efficacy of CMPP was characterized by *in vitro* and *in vivo* assays. As a result, this kind of magnetic polypyrrole nanocomposites were black spherical nanoparticles, possessing a favorable photothermal effect and the suitable particle size of 176.97 ± 1.45 nm with a chidamide loading rate of 12.92 ± 0.45%. Besides, comparing with PTT, CMPP exhibited significantly higher cytotoxicity and cellular apoptosis rate in two tumor cell lines (B16-F10 and HepG2). *In vivo* study, the mice showed obvious near-infrared (NIR) and magnetic resonance imaging (MRI) dual-modal imaging at tumor sites and sentinel lymph nodes (SLNs); on the other hand, magnetic targeting guided CMPP achieved a cure level on melanoma-bearing mice through preventing metastasis and thermotolerance. Overall, with high loading efficiency of chidamide and strong magnetic targeting to tumor sites and SLNs, CMPP could significantly raise efficiency of PTT by targeting tumor thermotolerance and metastasis, and this strategy may be exploited therapeutically to upgrade PTT with MPP as one of appropriate carriers for histone deacetylase inhibitors (HDACis).

## 1. Introduction

Photothermal therapy (PTT) is newly flourishing in clinical cancer treatment. It specifically damages tumor cells by delivering photothermal agents (PTAs) to tumor sites and raising the temperature through photothermal conversion (Zhou et al., 2012; Xiaoyang et al., 2018; Jing-Jing et al., 2018). With near-infrared (NIR) light irradiation, PTT generated a direct effect of hyperthermia or thermal ablation to ruin tumor cells (Qin et al., 2018; Chen et al., 2019; Cheng et al., 2020). At present, PTT has become one of the most effective means to treat cancer in clinical practice due to its spatiotemporal controllability, high specificity, minimal side effects, less invasiveness, and favorable compliance (Jing-Jing et al., 2018; Chen et al., 2019; Xinlong et al., 2021). In a typical PTT procedure, the light irradiation has to pass through healthy tissues without any side effects to generate heat in tumor tissue. Unlike ultraviolet and visible light, NIR light leads to much less damage after penetrating deep tissues (Weissleder, 2001; Vogel & Venugopalan, 2003; Gobin et al., 2007). Additionally, PTT exhibits favorable tissue selectivity due to the controllability of laser irradiation parameters (Liu et al., 2019; Haolan et al., 2021; Ping et al., 2021). PTT might increase the sensitivity of chemotherapy by destroying the integrity of cell membrane, resulting in the increase of local drug concentration (Fay et al., 2015). Although representing one of the most promising therapies in clinic, PTT has still been entangled with several challenges. The progressive thermotolerance of tumor cells hinders the therapeutic effect (Lihua et al., 2021; Xueqing et al., 2021); the retreat of PTT in convalescence causes the recurrence and metastasis of tumor, both leading to cancer deterioration and high mortality in clinic. Particularly, nonspecific targeting of PTAs leads to insufficient concentration in tumor sites and systematic toxicity (Jing-Jing et al., 2018). All above concerns call for an integrated strategy to advance PTT in reversing thermotolerance and metastasis.

Relevant research showed that the level of histone deacetylase (HDAC) in tumor tissues is decreased, leading to a rapid proliferation of tumor cells, then metastasis occurs (Yuhao et al., 2021). For the past few years, histone deacetylase inhibitors (HDACis) have demonstrated broad-spectrum anti-tumor effect by suppressing the activity of HDAC to activate the expression of anti-tumor genes and accelerate the differentiation and apoptosis of tumor cells (SM et al., 2016; Robert et al., 2021; Quinn et al., 2021). Thus, we assumed that HDACis could synergize PTT to counter lethal metastasis of tumors. For the moment, the combination of HDACi and PTT calls for an integrated drug delivery strategy with tumor-targeting and improving their solubility, cellular permeability, and tumor cells inhibition *in vivo* (Lei et al., 2018; Pengwei et al., 2021).

**Scheme 1.**
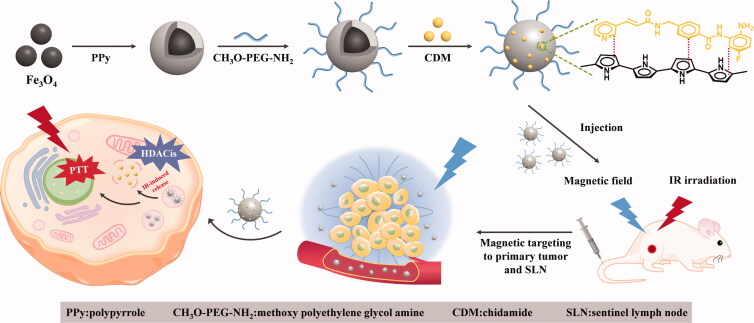
Schematic illustration of fabrication and anti-tumor mechanism of CMPP.

As much as possible to counter those concerns, we fabricated a magnetic-targeting nanocomposites encapsulating chidamide (CDM) to complement PTT for cancer treatment (Scheme 1), combining Fe_3_O_4_ nanoparticles (Fe_3_O_4_ NPs) and photothermal polymer material-polypyrrole. Briefly, pyrrole monomer and pyrrole-1-propionic acid were polymerized on the surface of Fe_3_O_4_ NPs to fabricate MPP, and CH_3_O-PEG-NH_2_ was grafted onto the surface to endow the nanocomposites better bioavailability and stealth owing to its long circulation property. At last, CDM was encapsulated into MPP to provide a novel chidamide-loaded magnetic targeting composite (CMPP) with NIR-responsiveness and magnetic targeting property for cancer theranostic.

MPP has ever been reported as a favorable PTT platform (Luo et al., 2020), herein we hypothesized the platform combining CDM could conduct a high-quality HDACis mediated PTT to counter thermotolerance and metastasis for visualized cancer PTT. CDM could conjugate polypyrrole firmly through π–π stacking and hydrophobic interaction to ensure the high loading rate of CDM, which guarantees the high level of accelerating the differentiation and apoptosis of tumor cells (Xue et al., 2017). Theoretically, CMPP actively targets to tumor sites and sentinel lymph nodes (SLNs) under magnetic field (Lili et al., 2016), then it could be endocytosed by tumor cells before PTT administration; and CDM is released into primary tumor sites and SLNs. Therefore, this PTT strategy with CMPP highlights the photothermal-chemotherapy through MRI visualized PTT in countering the thermotolerance and metastasis of cancer.

## 2. Material and methods

### 2.1. Materials

Iron (III) chloride hexahydrate (FeCl_3_·6H_2_O), sodium acetate (NaAc), sodium citrate tribasic dihydrate (Na_3_Cit·2H_2_O), sodium dodecylbenzene sulfonate (SDBS), N-[3-(dimethylamino) propyl]-N’-ethyl-carbodiimide hydrochloride (EDC), and N-hydroxysuccinimide (NHS) were supplied by Adamas Industrial Inc. (Shanghai, China). Pyrrole, Pyrrole-1-propionic acid, and methoxy polyethylene glycol amine (CH_3_O-PEG-NH_2_, Mw = 5000) were purchased from Aladdin Industrial Corporation (Shanghai, China).

### 2.2. Preparation of Fe_3_O_4_ @ PPy-PEG (MPP)

The preparation of Fe_3_O_4_ nanoparticles was described in Supplementary materials and Fe_3_O_4_ @ PPy (MP) was synthesized as follow. Briefly, 0.1 g Fe_3_O_4_ NPs and 1.8 g FeCl_3_·6H_2_O were dispersed in purified water (30 mL), stirring for 2.5 h at room temperature, then a mixture of SDBS solution (5.85 wt.%, 4 mL), pyrrole-1-propionic acid (0.03 g), and pyrrole (16 µL) was added into the water under 5 °C and kept stirring for 24 h. Finally, the crude product was washed with deionized water and methanol, respectively. The MP was obtained after vacuum drying.

Fe_3_O_4_ @ PPy-PEG (MPP) was prepared according to previous study (Yan et al., 2018). Briefly, MP (0.1 g) was dispersed into deionized water (20 mL), then EDC·HCl (0.1345 g) and NHS (0.0807 g) were added in sequence. The mixture solution was stirred under 4 °C for 50 min before CH_3_O-PEG-NH_2_ (0.03 g) was added into. The system kept stirring for 24 h and MPP was collected and washed with deionized water for three times.

### 2.3. Fabrication of CDM/Fe_3_O_4_ @ PPy-PEG (CMPP)

CDM was obtained according to our previous synthesis method (Ma et al., 2019). MPP (50 mg) was dispersed in tetrahydrofuran (THF) followed by the addition of CDM (10 mg) solution. The system was kept for stirring until THF completely volatilized. The product was washed with deionized water and collected by magnet, then CMPP was obtained by vacuum drying.

### 2.4. Characterization

Morphographies of Fe_3_O_4_ NPs, MP, and MPP were detected by transmission electron microscopy (TEM, TecnaiG2 F20 S-Twin; FEI, Hillsboro, OR). The hydrodynamic size was measured by dynamic light scattering (DLS, ZEN3600, Malvern Panalytical, UK). The fabrication of Fe_3_O_4_, MP, and MPP was verified by Fourier Transform Infrared Spectrometer (FT-IR, Nexus Model470, Nicolet, American). Crystal structure of Fe_3_O_4_ and MPP was determined by Wide Angle X-ray diffraction (XRD) while hysteresis loop was measured by Vibrating Sample Magnetometer (VSM, MicroSense, American).

### 2.5. Photothermal effect of MPP *in vitro*

Solution concentration and power intensity were two main factors affecting photothermal effect. Briefly, 2 mL MPP solution with different concentrations (0.1, 0.2, 0.4, 0.8, and 1 mg/mL) were exposure to 808 nm laser irradiation for 5 min (1.5 W·cm^−2^), and the temperature of MPP solution was recorded at predetermined time intervals; the influence of power density (0.5, 1, 1.5, and 2 W·cm^−2^) on photothermal effect was inspected using the aforementioned method with 1 mg/mL solution concentration. Purified water was set as control. All measurements were conducted in triplicate.

### 2.6. Determination of CDM loading efficiency and *in vitro* drug release

The determination of CDM loading efficiency was described in the Supplementary Materials. Besides, CDM released from CMPP with or without laser irradiation *in vitro* was conducted at different pH (7.4 and 5.2). Briefly, CMPP solution (2 mL) in a dialysis bag (MWCO: 3.5 kDa) was dialyzed against release media (40 mL, PBS: ethanol = 98:2, pH5.2, 7.4, respectively). At the beginning, the system was at 37 °C with or without exposure to 808 nm laser irradiation for 1 min at a power density of 1 W·cm^−2^. Then drawn 2 mL of dialysate at specific time intervals and replenished with equal volume of fresh release media at the same time. The absorbance of CDM was determined at 203 nm by UV–vis spectroscopy and the release amount of CDM was calculated using a predetermined standard profile (Figure S1).

**Figure 1.**
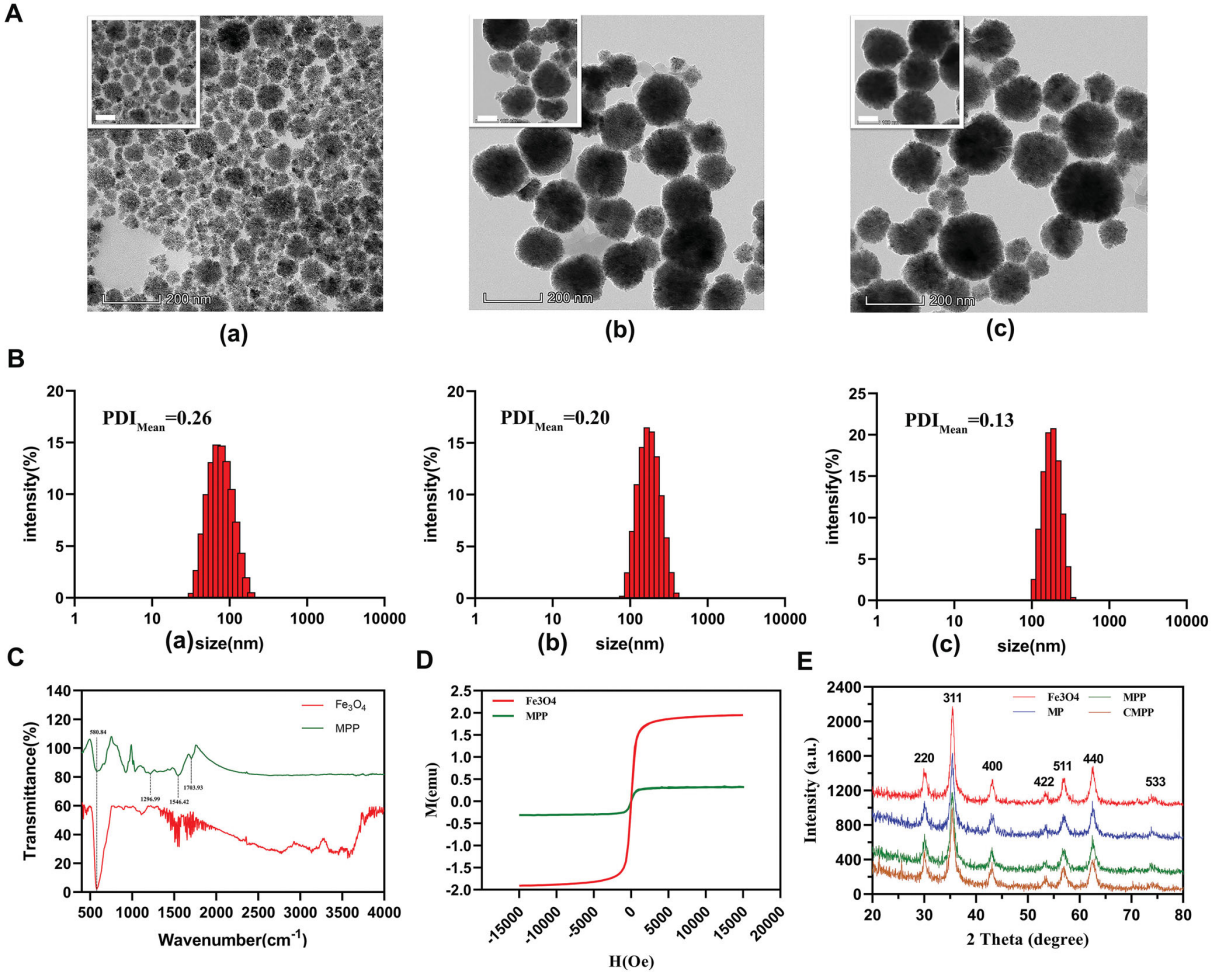
Characterization of Fe_3_O_4_, MP, and MPP. (A) TEM image of a) Fe_3_O_4_, b) MP, and c) MPP. Scale bar: 100 nm, 200 nm, respectively. (B) Hydrodynamic diameter distribution of a) Fe_3_O_4_, b) MP, and c) MPP by DLS, respectively. (C) FT-IR detection of Fe_3_O_4_ and MPP, respectively. Magnetic characteristics of Fe_3_O_4_ and MPP by (D)VSM and (E) XRD.

### 2.7. Cellular uptake assay

As cells with black pigments, B16-F10 cells pose huge interference to detecting fluorescence, so HepG2 cells were instead of B16-F10 to inspect the cellular uptake capacity of CMPP. Briefly, HepG2 cells (1 × 10^5^/mL) were seeded on confocal plates for 24 h, then free FITC-CDM or FITC-CMPP with a CDM concentration of 25 μg/mL was added to incubate for another 4 h. All cells in each plate were washed and fixed by 4% paraformaldehyde for 20 min before stained with 2-(4-amidinophenyl)-6-indole carbamidine dihydrochloride (DAPI). After that, the cells were collected and dispersed in 0.5 mL of PBS to observe under a confocal laser scanning microscope (CLSM, ZEISS710, Germany).

Cellular uptake assay was also probed by flow cytometer. Briefly, HepG2 cells (5 × 10^5^ cells/well) were seeded into 6-well plates and incubated for 24 h, then the culture medium was replaced with the formulation containing different concentrations of FITC-CDM (12.5, 25 μg/mL) or FITC-CMPP (the equal content of CDM with 12.5 and 25 μg/mL, respectively) and cultured for another 24 h. All cells were collected and detected by flow cytometry (BD FACS Calibur, San Jose, CA).

Magnetic targeting efficacy of CMPP was evaluated as below: HepG2 cells (2 × 10^5^/mL) were seeded on cover glass until cells adherence, two pieces of cover glass were put into a petri dish as a group. Negative group was treated with culture medium, M + CMPP group was treated with FITC-CMPP medium (the CDM concentration was 12.5 μg/mL) while one of the two glasses was interfered with magnet and the other was not. CMPP group was treated with FITC-CMPP medium (the concentration of CDM was 12.5 μg/mL) and CDM group was treated with FITC-CDM medium (the concentration of CDM was 12.5 μg/mL). All groups were cultured for 24 h. After that, all cells were collected and detected by flow cytometry.

### 2.8. *In vitro* cytotoxicity assay of MPP

CCK-8 assay was used to detect the biocompatibility of MPP against tumor cells B16-F10, HepG2, and normal healthy cells L929. Briefly, cell suspensions were seeded into 96-well plates with a density of 1 × 10^4^ cells per well. After incubating for 12 h, the medium was replaced with different concentrations of MPP mediums (4, 10, 20, 50, 100, and 200 μg/mL) and cultured for another 24 and 48 h. After that the fresh medium with 10% CCK-8 was added into each well and incubated for 2 h. Cell ability was quantified by a microplate reader (EL× 800; BioTek, Winooski, VT) at 450 nm. All measurements were conducted in triplicate.

### 2.9. *In vitro* photothermal-chemotherapy

The effect of photothermal-chemotherapy of CMPP against B16-F10 and HepG2 was tested by CCK-8 assays. Cell suspensions were seeded into 96-well plates with a density of 1 × 10^4^ cells per well. After incubating for 12 h, the medium was replaced with different concentrations of free CDM mediums (1.56, 3.13, 6.25, 12.5, 25, 50, 100, and 200 μg/mL) and CMPP mediums (the content of CDM was the same to free CDM) and cultured for 12 h. For B16-F10 cells, laser groups exposed to 808 nm laser irradiation for 1, 3, and 6 min at a power density of 25 mW·cm^−2^, respectively. Then continue to cultivate until to 24 h, while without laser groups were the same to laser groups but irradiation. For HepG2 cells, the method was the same above except the power density was 0.597 W·cm^−2^, the reason was that with black pigments, B16-F10 cells made a certain impact on near infrared absorption, so the density on B16-F10 cells was weaker than that of HepG2 cells. After that the fresh medium with 10% CCK-8 was added into each well and incubated for 2 h. Cell ability was quantified by a microplate reader (EL× 800; BioTek, Winooski, VT) at 450 nm. All measurements were conducted in triplicate.

### 2.10. Apoptosis assay

HepG2 cells were chosen to evaluate the apoptosis induced by CMPP for the reason of the pigments of B16-F10. Briefly, HepG2 cells were seeded into 6-well plates (5 × 10^5^ cells/well) and incubated for 24 h, then the culture medium was replaced with the formulation containing free CDM (25 μg/mL), CMPP (the concentration of CDM was 25 μg/mL) and MPP (an equivalent concentration to CMPP) with or without 808 nm laser irradiation (0.597 W·cm^−2^, 3 min/well). After incubating for 24 h, all cells were collected to investigate the apoptosis with FITC-AnnexinV/PI by flow cytometry.

### 2.11. Western blotting

In order to measure the expression level of Histone H3 after treated with CMPP on B16-F10 cells, western blotting experiment was performed. Briefly, B16-F10 cells in complete RPMI (2 mL) were seeded into 6-well plates (1 × 10^6^ cells per well) and cultured for 12 h. Then the original medium in each well was replaced by the formulations (CDM and CMPP) in RPMI (the CDM concentration was 25 μg·mL^−1^). After incubation for 4 h, the cells were washed and RIPA reagent (300 μL) was added to each well. Afterwards, the cells were collected by scraping. This whole process was performed in an ice box. The proteins extracted from the cells were measured by the western blotting experiments.

### 2.12. *In vivo* biodistribution

In this study, all animals were treated in accordance with the Guiding Principles for the Care and Use of Laboratory Animals, the Second Military Medical University and animal procedures were approved by the Institutional Animal Care and Use Committee of the Second Military Medical University.

In order to evaluate biodistribution of CMMP *in vivo*, B16-F10 cells (1 × 10^5^ cells per mouse) were subcutaneously injected into C57BL/6 mice to prepare melanoma-bearing mice models. When the tumor volume reached about 100 mm^3^, mice were given CMPP (4 mg CDM per kilogram) by tail intravenous injection and divided into two groups. M + group was treated with magnetic field for 30 min by putting a Neodymium magnet near the tumor site, while M-group without that. After injection for 8 h, the mice were sacrificed, and the main organs (the heart, liver, spleen, lungs, and kidneys) and tumors were collected to detect the content of Fe^3+^ through Prussian blue dyeing. The result was quantified by Image-J software (National Institutes of Health, Bethesda, MD).

### 2.13. *In vivo* magnetic resonance imaging (MRI)

To evaluate the magnetic targeting efficacy of CMPP, MIR scanner (ASPECT M7 Imaging system) was applied as follows. The mice were divided into two groups including M + CMPP + group and CMPP + group (‘M+’ meant with the intervention of magnetic field; ‘+’ meant exposure to NIR irradiation). All mice were treated with CMPP (4 mg CDM per kilogram) through tail intravenous injection, after that M + CMPP + group was under magnetic field (a Neodymium magnet) for 40 min while CMPP + group without that, then these two groups were under NIR irradiation (1.5 W·cm^−2^) for 5 min. The detection was taken by MIR scanner before injection and after injection for 40 min, respectively.

### 2.14. *In vivo* photothermal therapy

C57BL/6 mice with melanoma-bearing were divided into three groups randomly, each group was treated as follows. M + CMPP + group was given CMPP (4 mg CDM per kilogram) by tail intravenous injection with the intervention of magnetic field (a Neodymium magnet) for 40 min before exposure to 808 nm laser irradiation (1.5 W·cm^−2^, 5 min). CMPP + group was the same to M + CMPP + group but without exposure to 808 nm laser irradiation. Saline group (negative group) was the same to CMPP + group but CMPP was replaced by saline. The photos were recorded at intervals using an IR infrared camera (Thermal Intelligence, 323 Pro, FOTRIC).

### 2.15. *In vivo* antitumor effects

Tumor-bearing C57BL/6 mice were randomly divided into six groups (*n* = 6 per group) and treated as follows. Group 1: Saline (control), group 2: free CDM, group 3: CMPP, group 4: MPP+, group 5: CMPP+, group 6: M + CMPP+ (‘+’ meant exposure to 808 nm laser irradiation; ‘M+’ meant with the intervention of magnetic field). Then the formulations above injected intravenously through the tail vein (the amount of CDM in each group was 4 mg·kg^−1^). After injection, the treatment of each group as following: groups 4 and 5 were exposed to 808 nm laser irradiation with a power density of 1 W·cm^−2^ for 1 min (each mouse); group 6 interfered with magnetic field (a Neodymium magnet) for 5 min and then exposure to 808 nm laser irradiation for 1 min (each mouse). During the experiment, the length and width of tumors were measured to calculate the volume (length × width^2^/2) and the body weight were recorded.

### 2.16. Histological analysis

The tumor tissues and lungs were excised from the mice after 12 d treatment, and fixed in 4% formaldehyde solution before embedded in paraffin following by cutting into sections. Afterwards, samples were tested with hematoxylin-eosin staining (H&E) and immunofluorescent staining (Histone H3, HSP70).

### 2.17. Statistical analysis

Statistical significance was analysis by statistical computation using SPSS version 23.0 (SPSS Inc., Chicago, IL). Normality testing was performed to assess the variable distribution. One-way ANOVA was used between three or more groups when the data distributing normally. Data were presented as mean ± standard deviation (SD). The significant level was set as *p*<.05.

## 3. Results and discussion

### 3.1. Preparation and characterization of CMPP

Fe_3_O_4_ NPs, Fe_3_O_4_-PPy (MP), and Fe_3_O_4_-PPy-PEG (MPP) were provided as black powders after vacuum drying. The morphologies and sizes of these three magnetic nanoparticles were investigated by TEM and DLS, respectively. TEM revealed these magnetic nanoparticles were favorable spherical, and polypyrrole layer was evenly coated on the surface of Fe_3_O_4_ nanoparticles (Figure 1(A)). As shown in Figure 1(B), the hydrodynamic size of Fe_3_O_4_, MP, and MPP was 72.72 ± 1.30, 159.20 ± 13.95, and 176.97 ± 1.45 nm, respectively, further indicating polypyrrole grafting and entangling on the surface of Fe_3_O_4_, which endows the system photothermal conversion capacity. Besides, these three nanoparticles’ dispersion indexes (PDIs) were 0.26 ± 0.21, 0.20 ± 0.17, and 0.13 ± 0.02, respectively, indicating all the nanoparticles could be well dispersed in the aqueous solution. The decrease of PDI also showed that the hydrophilicity of the carrier was improved owing to the introduction of CH_3_O-PEG-NH_2_, resulting in the complex more stable in aqueous solution.

Modification of Fe_3_O_4_ was verified by FT-IR as shown in Figure 1(C). It indicated Fe_3_O_4_ had a special absorption peak at the wavenumber of 580.84 cm^−1^, which reflected the absorption of Fe-O; in addition, the wavenumber of 1703.93, 1546.42, and 1296.99 cm^−1^ affiliated to amide band I, amide band II, and amide band III, respectively, further hinting the entanglement of PEG on the surface of MP.

Superparamagnetic Fe_3_O_4_ nanoparticles have been widely used in industry and scientific research due to their unique physical properties. Herein, VSM is utilized to investigate the magnetic characteristics of Fe_3_O_4_ and MPP nanomaterials, and the results showed both Fe_3_O_4_ and MPP presented superparamagnetic property with almost zero coercivity and remanent magnetism, though magnetic saturation intensity of MPP nanoparticles (10.5 emu/g) is much lower than that of Fe_3_O_4_ nanoparticles (64 emu/g) (Figure 1(D)).

The absorption peaks were examined by large-angle XRD. The data indicated that Fe_3_O_4_ nanoparticles had a cubic crystal structure. As shown in Figure 1(E), the height of each peak in the XRD pattern decreased with the increase of coating layers. The four substances had diffraction peaks at the corresponding main absorption peak positions (220), (311), (511), and (440), and there was no left or right deviation, indicating the lattice parameters and unit cell of Fe_3_O_4_ in each nanoparticle did not change during the procedure. In addition, the intensity of absorption peak such as (311) was significantly attenuated from 1000 to 700 a.u., hinting CDM successfully encapsulated into MPP reduced the absorption peak of the iron core.

### 3.2. *In vitro* photothermal effect

**Figure 2.**
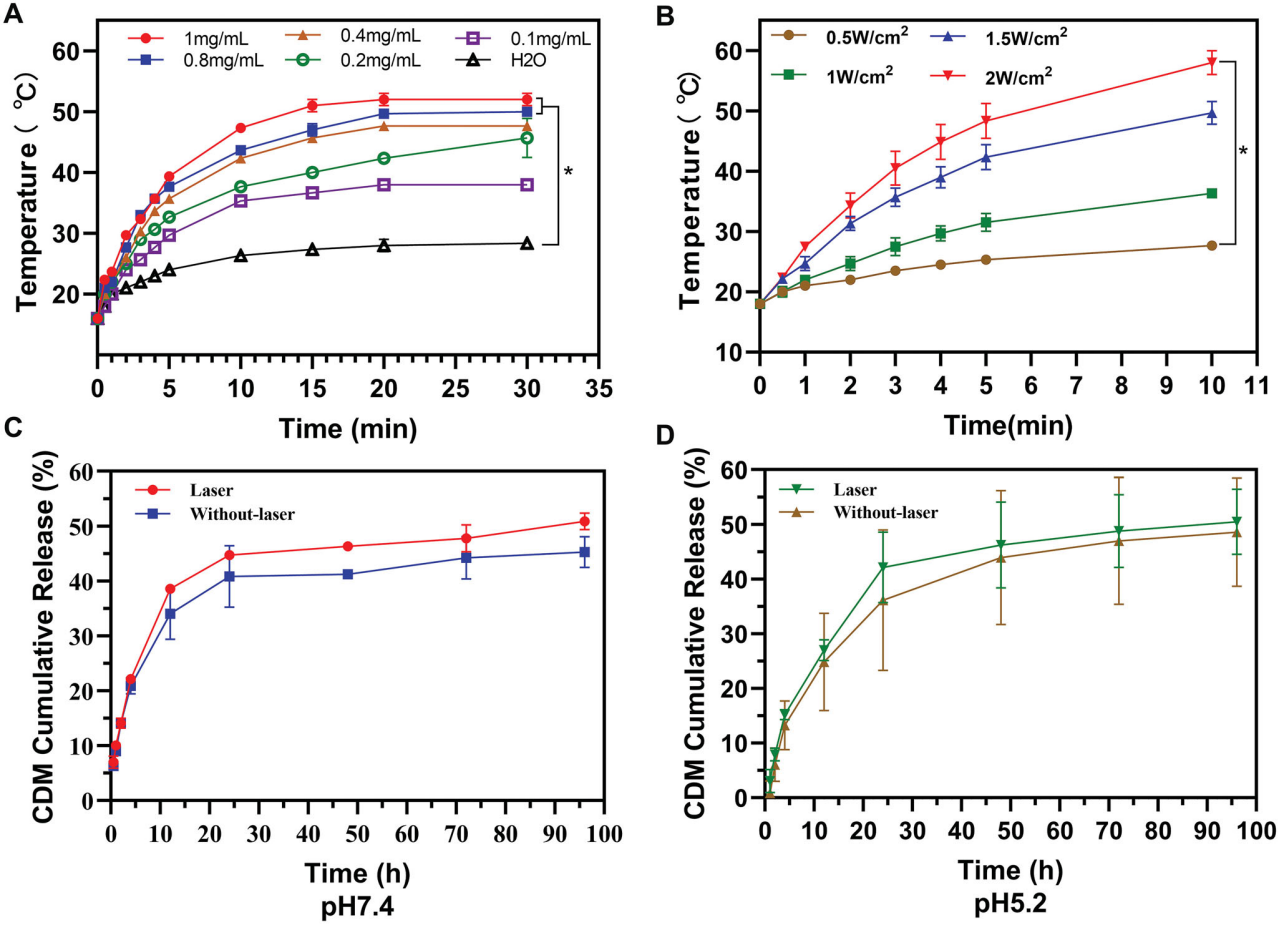
Influence factors of photothermal effect of MPP and drug release. Influence factor of suspension concentration (A) and laser power density (B). Drug release from MPP *in vitro* at pH 7.4 (C) and pH 5.2 (D).

The concentration of PTA and the laser power density were the key factors for evaluating PTT. Generally, the temperature of MPP suspensions increased more or less with concentration or laser power density increasing. When exposed to 808 nm laser irradiation (1.5 W· cm^−1^) for 5 min, the temperature of 1 mg/mL MPP suspension reached to 52 ± 1 °C, which could cause severely damage to tumor cells locally. By comparison, the temperature of 0.1 mg/mL MPP suspension reached to 38 ± 0 °C and that of water was only increased by 12.3 ± 0.6 °C from 16 ± 0 °C to 28.3 ± 0.6 °C (Figure 2(A)). Importantly, when the laser power density increased, the temperature of MPP (1 mg/mL) suspension increased significantly (*p*<.05) (Figure 2(B)). All the data above manifest favorable photothermal conversion efficacy of CMPP deriving from MPP.

### 3.3. CDM-Loading efficiency and drug release *in vitro*

As a combining strategy, chemo-PTT could promote drug delivery into tumors as the generated hyperthermia (Juan et al., 2018; Yihan et al., 2021; Zheng et al., 2021). Another reason why polypyrrole could significantly increase drug delivery efficiency is the π-π stack bond between CDM and polypyrrole. As shown in Table S1, due to the strong interaction between polypyrrole and CDM by π-π stack bond, CMPP has a much higher drug loading rate (12.92 ± 0.45%), as compared to Fe_3_O_4_@nSiO_2_ (an iron core photothermal carrier previously reported in our group, the drug-loading efficiency was only 3.4%) (Zhan et al., 2017; Anh et al., 2021).

CDM release from MPP was investigated at different pH conditions (pH 7.4 or 5.2) within (1 min) or without NIR irradiation. The results showed the accumulative release of CDM at pH 7.4 was similar to pH 5.2 with or without NIR irradiation after 48 h (46.35 ± 0.37% *vs.* 41.30 ± 0.19% at pH 7.4, 46.28 ± 7.87% *vs.* 43.97 ± 12.26% at pH 5.2), indicating CDM could be released either in neutral environment (pH 7.4) or tumor microenvironment (minimum pH 5.2). Meanwhile, under the stimulated condition of NIR laser irradiation, the release of CDM was further promoted to 44.78 ± 0.07% in 24 h while it was only 40.85 ± 5.62% without irradiation at pH 7.4 (at pH5.2, the release amount is 42.17 ± 6.47% with laser and 36.18 ± 12.84% without laser) (Figure 2(C,D)), the result indicated NIR irradiation could promote the release of CDM into the cytoplasm more or less as a result of local hyperthermia which reduce the interaction between the MPP and CDM.

### 3.4. Cellular uptake of CMPP

**Figure 3.**
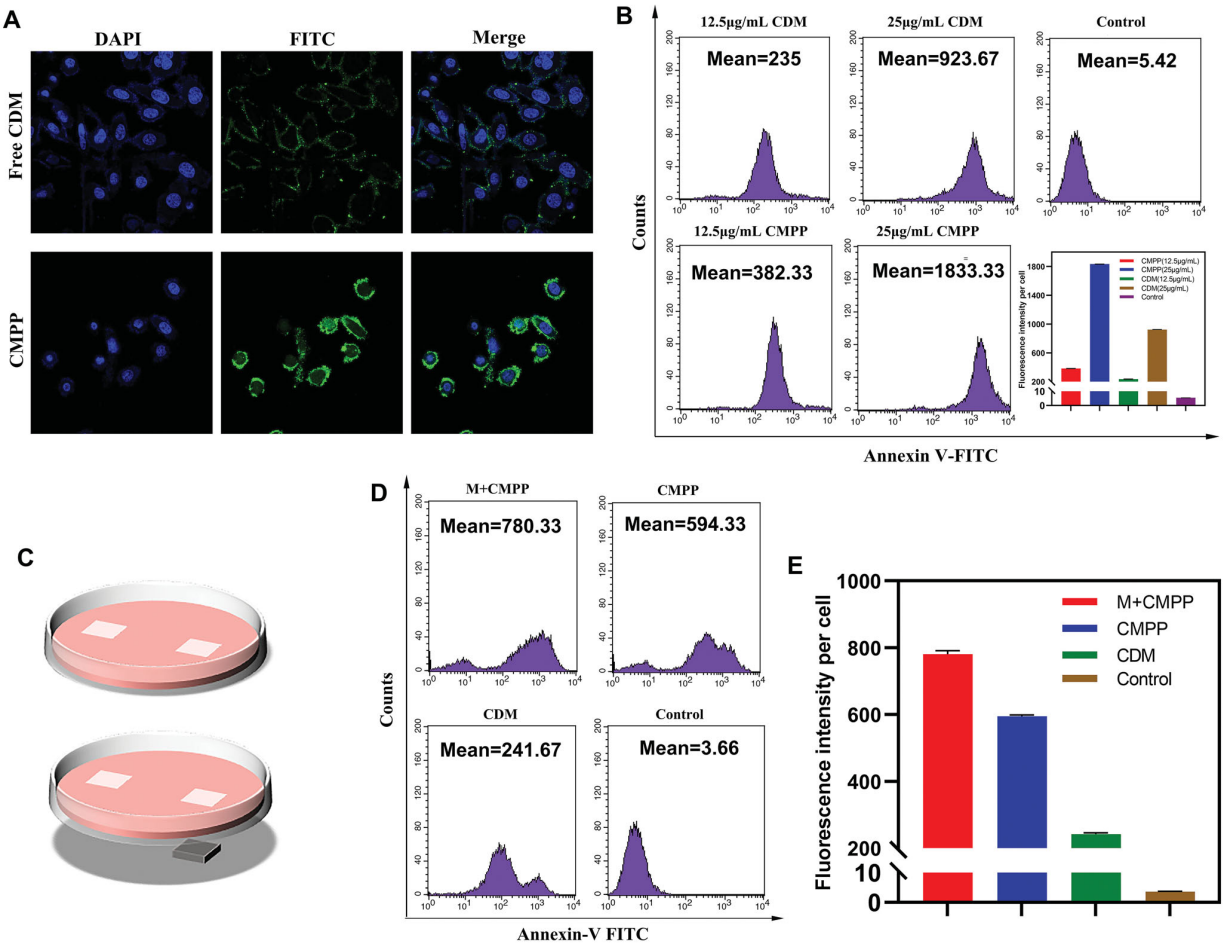
Cellular uptake studies on HepG2. (A) Cellular uptake assays by laser confocal microscopy and (B) fluorescent microscope. (C) Illustration of CMPP with (down)/without (up) the intervention of magnetic field. (D) Magnetic targeted cellular uptake under fluorescent microscope and (E) quantification.

A high efficiency of intracellular uptake is primary premise for drug delivery system. Cellular uptake of CMPP in HepG2 cells was estimated by confocal laser scanning microscopy (CLSM). The resultant images (Figure 3(A)) revealed the fluorescence intensity (green) treated with CMPP is much higher than that of free CDM, probably because the positive charge of protonated secondary amine of pyrrole segments at acidic pH (tumor microenvironment pH) demonstrated a better affinity with negative charged cytomembrane, reinforcing the cellular uptake level.

Cellular uptake in HepG2 was further quantitatively investigated by flow cytometry. As shown in Figure 3(B), when CDM concentration was 25 μg/mL, the fluorescence intensity of CMPP (1833.33 ± 2.08) was much higher than that of free CDM (923.67 ± 1.53), which was in accordance with the result of CLSM above.

The magnetic targeting capacity of CMPP was further evaluated, and the graphic illustration was shown in Figure 3(C). The results (Figure 3(D,E)) showed that with CDM concentration was 12.5 μg/mL, the average fluorescence intensity of M + CMPP was 780.33 ± 10.97 per cell, while that of CMPP and CDM were 594.33 ± 4.51 and 241.67 ± 4.51, respectively. These results validated that the amount of engulfed CMPP by HepG2 cells was much higher than that of free CDM and could be further strengthened in a magnetic field.

### 3.5. *In vitro* cytotoxicity study

**Figure 4.**
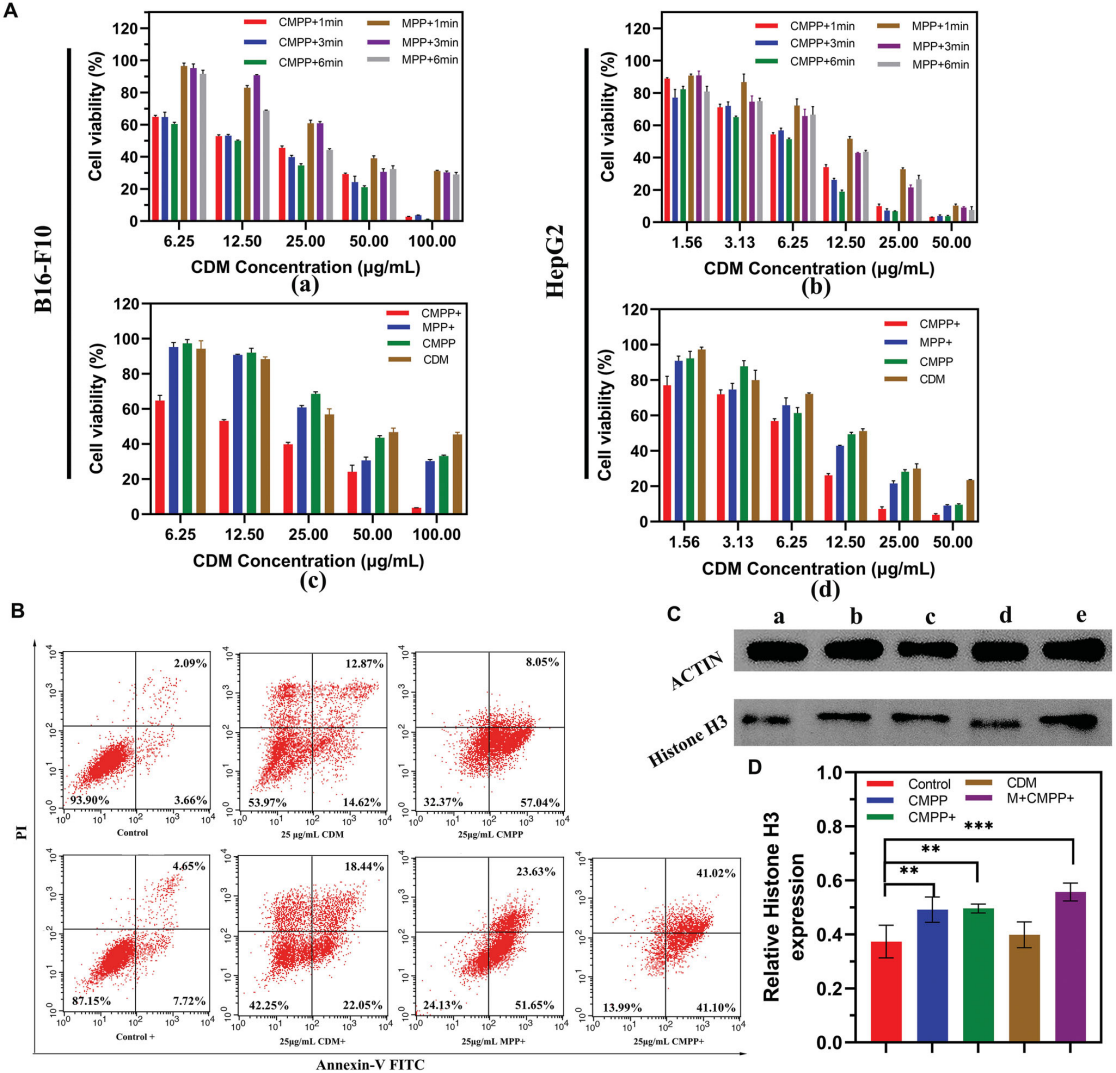
The efficiency and mechanism of CMPP for tumor cells. (A) Cytotoxicity of CMPP and MPP with different irradiation time (up) on a) B16-F10, b) HepG2 and different treatments (down) on c) B16-F10, d) HepG2. (B) Cell apoptosis by flow cytometry flow. (C) Western blotting analysis and (D) quantified Histone H3 expression levels in B16-F10 cells after treatment for 4 h. (a) Control; (b) CMPP; (c) CMPP+; (d) CDM; (e) M + CMPP+.

Cytotoxicity of MPP was evaluated via different formulations on B16-F10, HepG2, and L929 cells, the result (Figure S2) suggested that the relative viabilities of all cells were above 90%, even though the concentration of MPP was up to 200 μg/mL, indicating MPP has favorable biocompatibility as the PTT agent. Subsequently, B16-F10 and HepG2 cells were used to assess the efficiency of photothermal-chemotherapy. As shown in Figure 4(A), the relative cell viability generally exhibited a concentration-dependent tendency. Furthermore, compared with control group, the cell growth was significantly inhibited. Specifically, the viability of cells treated with CMPP under NIR irradiation was much lower than that of MPP at all-time points (Figure 4A(a,b)), indicating CDM exerting cell inhibition effect in the PTT procedure. Also, it was obvious that CMPP + group (cells treated with CMPP and NIR irradiation) showed higher mortality compared to those treated with MPP+, MPP, or CDM in both cell lines (Figure 4A(c,d)). Above all, MPP displayed favorable biocompatibility without NIR irradiation meant good biosafety for clinical use. Besides, the relative cell viability of CMPP + group was lower than that of MPP + group at the corresponding concentration, demonstrating the synergistic therapeutic effect of HDACi and PTT.

Apoptosis assay was conducted to analyze the antitumor effect of CMPP. With NIR irradiation, CMPP, MPP, and CDM (the content of CDM of all groups were equal with 25 μg/mL; the content of MPP was equivalent to the MPP content of CMPP group) caused HepG2 cells apoptosis in a dose-dependent manner compared to control group. Specifically, CMPP showed an apoptotic cell proportion of 82.12%, which was higher than that of MPP (75.28%) and free CDM (40.49%); in addition, without the treatment of NIR irradiation, CMPP showed an apoptotic cell proportion of 65.09%, higher than that of free CDM (27.49%) (Figure 4(B)), as well as indicating CMPP could significantly enhance the therapeutic effect.

To verify the efficacy of HDACi in CMPP, the expression of Histone H3 in B16-F10 cells was studied by western blot analysis with actin as a control group (Figure 4(C,D)), all groups had equivalent amount of CDM. The relative expression of Histone H3 in cells with M + CMPP + was 0.56 ± 0.03, while that of CMPP+, CMPP, CDM, and Control groups were 0.49 ± 0.04, 0.49 ± 0.04, 0.40 ± 0.04, and 0.37 ± 0.06, respectively, demonstrating that with the intervention of magnetic field, CMPP could massively inhibit the activity of HDAC, thus up-regulate the expression of Histone H3 significantly (*p*<.001), providing premise for supplementing polypyrrole mediated PTT.

### 3.6. *In vivo* magnetic resonance imaging and biodistribution

**Figure 5.**
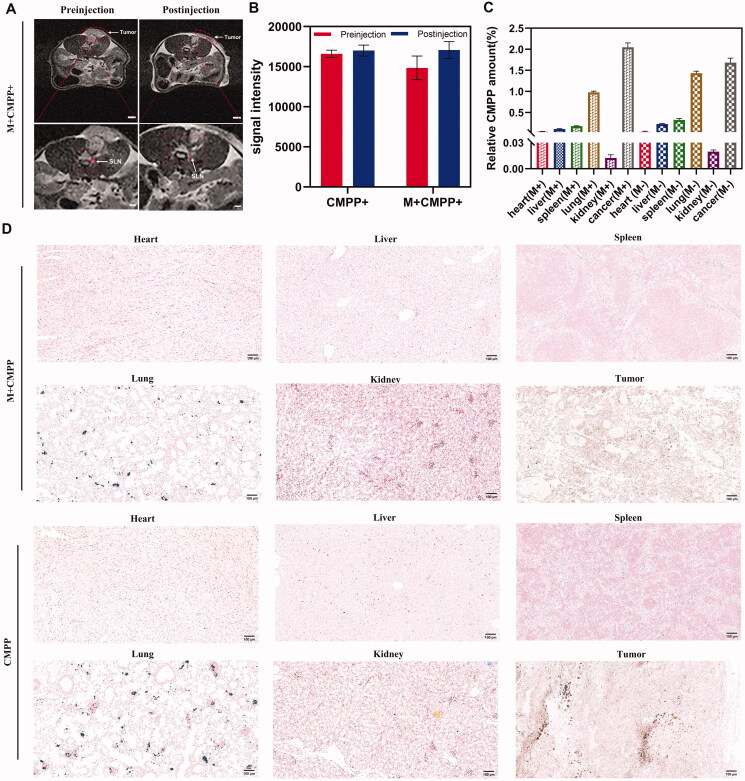
MRI capability and tissue distribution of CMPP *in vivo*. (A) Magnetic resonance imaging of CMPP, scale bar: 250 mm (up), 100 mm (down); (B) quantified signal intensity before and after injection. (C) Quantified Fe^3+^ expression levels after treatment for 8 h and (D) biodistribution of CMPP in major organs and tumor, scale bar:100 μm.

Magnetic targeting of CMPP *in vivo* was verified by MRI and images were compared between pre- and post-injection with/without the intervention of magnetic field for 40 min in each group. Neodymium-iron-boron magnet was used to simulate the magnetic field effect at the tumor site during the experiment, resulting in non-solid melanoma malformation as the figures showed (Figure 5(A)). The signal intensity of tumor sites in mice with/without magnetic field intervention significantly different. Briefly, the relative signal intensities of all groups were increased, reflecting the T1 enhancement effect after injection for 40 min. Therein, the relative signal intensity of M + CMPP + group increased by 14.77 ± 4.63%, while that of CMPP + group only increased by 2.44 ± 1.52% (Figure 5(B)), denoting more CMPP could target to tumor sites under the intervention of external magnetic field. Here, because of its small enough particle size, CMPP exhibited T1 enhancement effect rather than T2 enhancement effect (Lu et al., 2017).

Lymphatic metastasis is one of the main spreading routes of cancer cells. In the early stage of metastasis, the SLNs are usually the main metastasis hosts of tumor cells (Lili et al., 2016; Hansine et al., 2021). In this study, the guidance of multi-mode images was utilized to determine the location of lymphatic metastasis and optimize the cancer treatment. As shown in Figure 5(A), guided by MRI image, the SLNs around the primary tumor became clear visible, which facilitated the diagnosis of CMPP. The results indicated, after injection for 40 min, CMPP transferred from primary tumor to SLNs. Correspondingly, with the intervention of magnetic field and NIR, SLN images significantly shrank, indicating visualized PTT could induce thermal ablation of primary and metastatic tumor cells in SLNs to prevent the distal metastasis in the MRI visual field.

Biodistribution and accumulation of CMPP were evaluated by Prussian blue staining test. After 8 h intravenous injection, CMPP exposed to magnetic field could be effectively accumulated in tumor tissues with a rate of 2.04 ± 0.11%, which was higher than that in heart (0.04 ± 0.01%), liver (0.10 ± 0.01%), spleen (0.17 ± 0.01%), lungs (0.97 ± 0.03%), and kidney (0.01 ± 0.004%); besides, without the intervention of magnetic field, the accumulation rate of CMPP targeting to tumor was 1.68 ± 0.11% (0.04 ± 0.01% in heart, 0.22 ± 0.01% in liver, 0.32 ± 0.03% in spleen, 1.43 ± 0.05% in lungs, 0.02 ± 0.002% in kidney), as shown in Figure 5(C,D). The result indicated CMPP could be precisely targeted to tumor site to reinforce the synergistic therapeutic effect when exposed in magnetic field.

### 3.7. *In vivo* photothermal imaging

**Figure 6.**
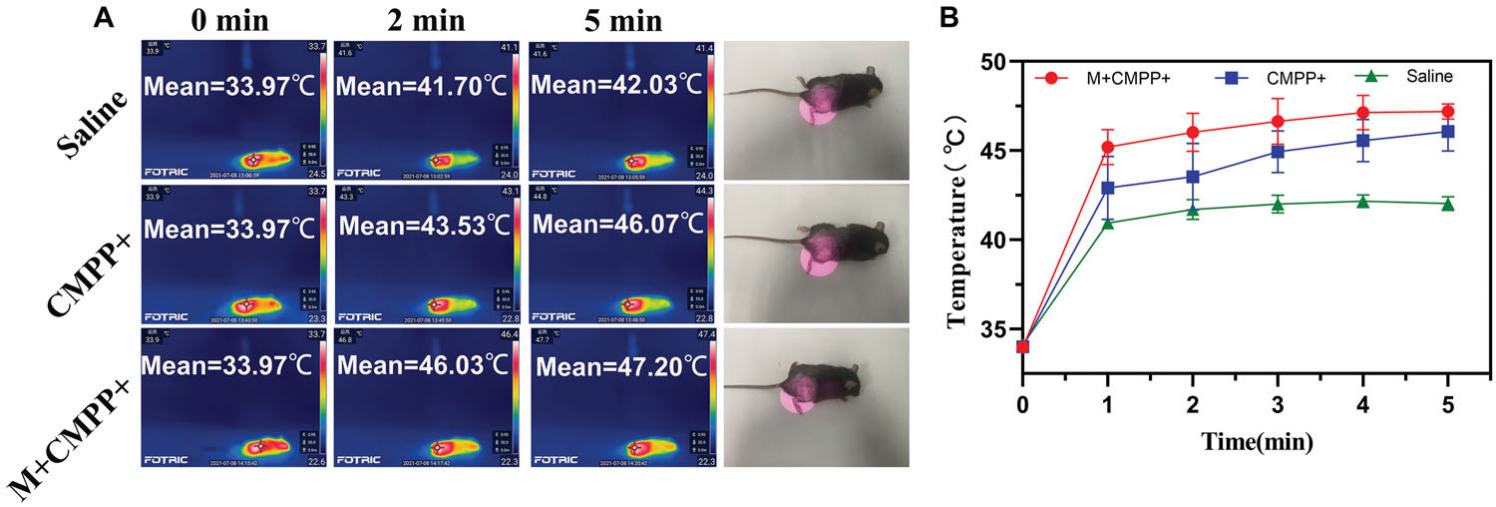
Photothermal effect of CMPP *in vivo* with or without magnetic field intervention. (A) Photothermal imagining in 5 min and (B) quantitation of temperature at certain intervals.

Infrared imaging experiment *in vivo* was conducted to further investigate the photothermal and magnetic targeting property of CMPP. With the exposure to 808 nm laser irradiation at tumor site for 5 min, the temperatures of M + CMPP + group rapidly increased by 13.23 ± 0.51 °C from 33.97 ± 0.11 °C to 47.20 ± 0.44 °C, demonstrated much higher temperature increase than those of CMPP + and saline group (from 33.97 ± 0.11 °C to 46.07 ± 1.10 °C and 42.03 ± 0.38 °C, respectively) (Figure 6). Through the gap of temperature between M + CMPP + group (47.20 ± 0.44 °C) and CMPP + group (46.07 ± 1.10 °C) was small, the potential vital PTT mechanisms of these two groups are different, leading to a prominent photothermal effect for cancer of M + CMPP + group. The mechanism of PTT includes hyperthermia (Odion et al., 2021; Granja et al., 2021) and thermal ablation (Zhang et al., 2021; Shan et al., 2021). Therein, hyperthermia refers to the phenomenon that tumor cells necrosis when they are directly exposed to the temperature range from 41 °C to 47 °C. Owing to the heterogeneity of the blood vessels at the tumor site, the tumor being more sensitive to high temperatures (41–47 °C) than normal tissue. When the temperature in tumor site raised beyond 47 °C, the process known as thermal ablation occurs, mainly causing severe cell membrane damage, cell material outflow, cell protein degeneration, DNA damage, and complete inactivation of cancer cells (Guorong et al., 2021; Miao et al., 2021). Therefore, an evolution from hyperthermia to thermal ablation could explain the results that magnetic field guided CMPP resulted in a stronger efficacy of PTT.

### 3.8. *In vivo* anticancer effect

**Figure 7.**
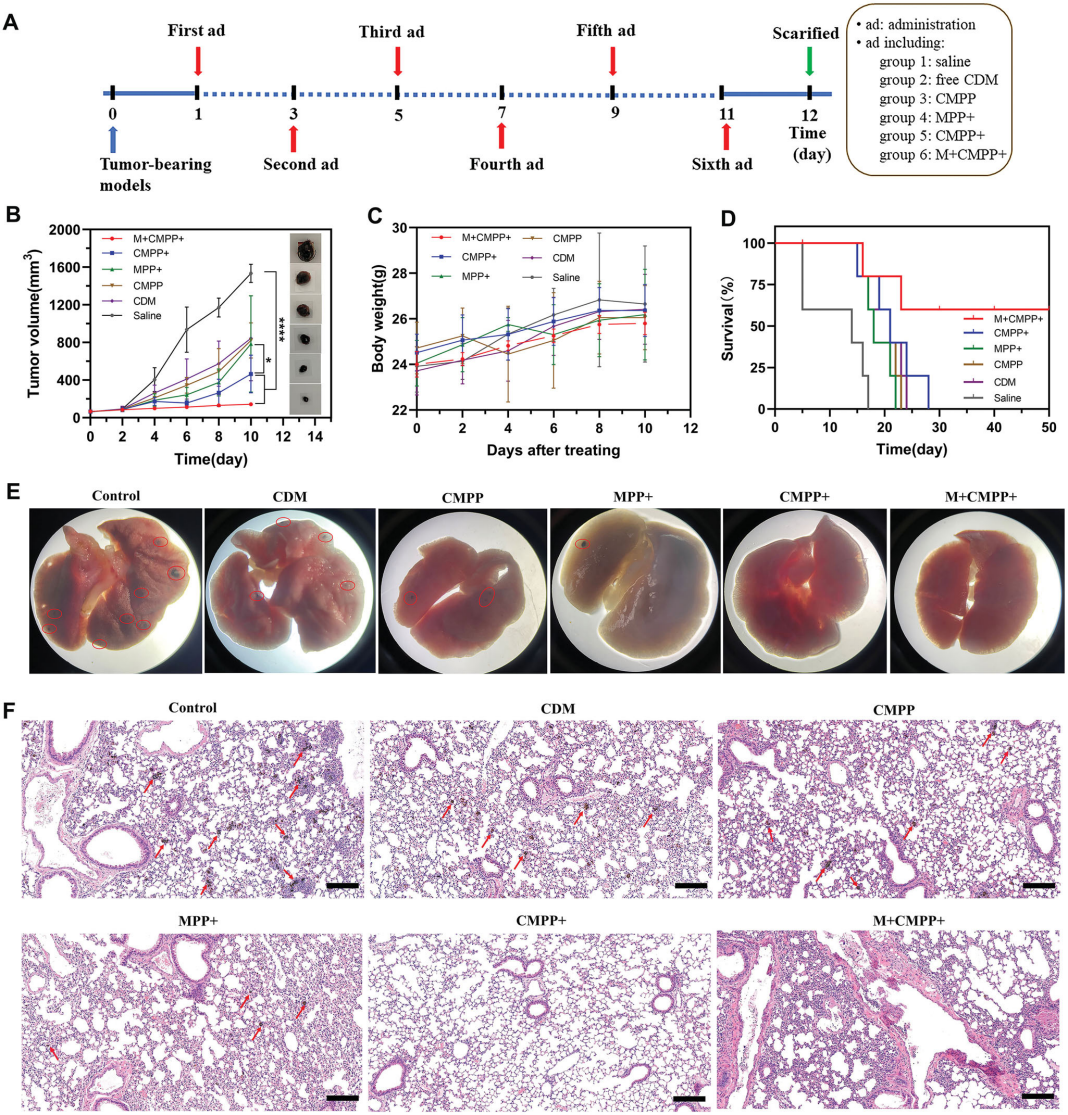
*In vivo* antitumor studies of CMPP. (A) Illustration of time schedule *in vivo*; (B) Tumor growth curve of mice with different treatment and representative images; (C) body weight changes with time; (D) morbidity free survival curves; (E) the photographs of lung tissues with the first dead mice bearing B16-F10 melanoma in each group after treatment; (F) H&E staining of the lung sections from the first dead mice bearing B16-F10 melanoma in each group after treatment. Scar bar: 200 μm, the red circles, and arrows represented melanoma.

The anticancer effect of CMPP *in vivo* was conducted on tumor-bearing C57BL/6 mice models, and the time schedule was shown in Figure 7(A). Compared with saline group, tumor growth was significantly inhibited (*p*<.0001) with the treatment of M + CMPP + over the entire trial period (Figure 7(B)). Specifically, it showed that the tumor volume in CMPP + and MPP + groups had significant difference (*p*<.05), indicating CDM loaded in MPP could exert an obvious therapy to cancer. Besides, the treatment of CMPP + resulted in a significant slower rate of tumor volume growth compared to control group (*p*<.0001). At the same time, no significant weight change was observed in all treatment groups, verifying the favorable biocompatibility of the nanoplatform (Figure 7(C)). The survival time of mice in M + CMPP + group was obviously prolonged compared to other groups, manifesting the treatment of M + CMPP + prevented the growth and metastasis of melanoma (Figure 7(D)).

### 3.9. Histological analysis

After 12 d’ treatment, one alive mouse in each group was sacrificed, then lungs and tumor were excised to detect through HE staining and immunofluorescent staining (Histone H3, HSP70). As shown in Figure 7(E), lung metastases of melanoma were absent in the groups treated with M + CMPP + and CMPP+, while other groups demonstrated pulmonary melanomas metastases, corresponding to the result of HE staining (Figure 7(F)).

**Figure 8.**
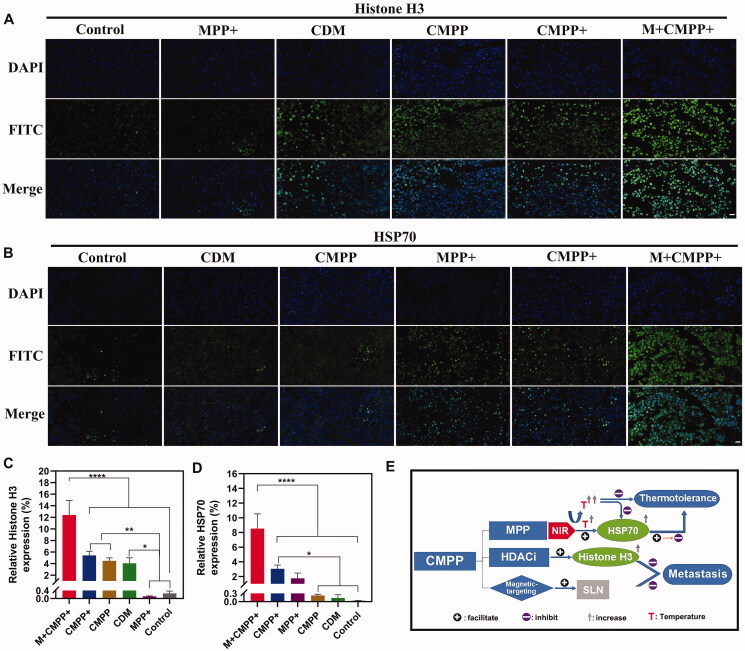
Immunofluorescence staining analysis and flow chart of antitumor mechanism of CMPP. (A) Immunofluorescence staining analysis on Histone H3 (blue: cell nucleus; green: Histone H3) and (B) HSP70 (blue: cell nucleus; green: HSP70). Scar bar: 20 μm. The quantified expression levels of (C) Histone H3 and (D) HSP70 after immunofluorescence staining by image €(E) The antitumor mechanism of CMPP *in vivo*.

For immunofluorescent staining, it was clear that the expression levels of H3 and HSP70 in M + CMPP + group were significantly increased compared with other groups (Figure 8(A–D)), suggesting the photothermal therapy and chemotherapy simultaneously played their role, respectively, to achieve the CDM mediated PTT, corresponding to the result of western blot in Figure 4(D). When CMPP was targeted to tumor cells with the intervention of magnetic field, NIR irradiation led to the temperature rising in tumor tissue, on the one hand, HSP70 was generated to help tissue tolerating the heat atmosphere. However, with continually irradiated by NIR, the accumulated heat would destruct tumor cells, leading to a decease expression of HSP70. Meanwhile, HDACi was released by the drive of heat, leading to the increase of Histone H3, and tumor cells metastasis was inhibited. Additionally, in the magnetic field, more CMPP aggregated to the SLNs and inhibited metastasis of cancer, thus the lung metastasis of melanoma could be further retarded (Figure 8(E)).

## 4. Conclusions

In summary, with the aim to verify the assumption that HDACis could complement PTT to reverse tumor thermotolerance and metastasis, a magnetic polypyrrole nanocomposite loading CDM was fabricated as a visualized photothermal-chemotherapy for cancer. Under the magnetic field and NIR irradiation *in vitro*, this nanocomposite showed a satisfactory photothermal effect. In cellular level, MPP exhibited good biosafety and CMPP could significantly inhibit the vitality of tumor cells. *In vivo*, such administration achieved dual-modal imaging with the intervention of NIR and MRI, and tumors were significantly inhibited and the survival period was greatly prolonged. A striking result was that several tumors were even cured and melanoma pulmonary metastasis was effectively suppressed, which significantly due to the therapeutic effect of CMPP to sentinel lymph nodes and the increase of histone acetylation. Therefore, this research not only exhibited the combination of HDACi and PTT reversing thermotolerance and metastasis, but also may be exploited therapeutically to upgrade PTT with MPP as one of appropriate carriers for HDACis.

## Supplementary Material

Supplemental Material
