# Supplementary material for: Chidamide stacked in magnetic polypyrrole nano-composites counter thermotolerance and metastasis for visualized cancer photothermal therapy
Source: Drug Deliv. 2022 Apr 27;29(1):1312–25. doi: 10.1080/10717544.2022.2068697 (PMC9067950; doi:10.1080/10717544.2022.2068697)
Supplement: Supplemental Material [file IDRD_A_2068697_SM0199.docx]

**Supplementary materials**

**Preparation of Fe_3_O_4_ nanoparticles (Fe_3_O_4_ NPs)**

Fe_3_O_4_ NPs were prepared by solvothermal method^41,42^. Briefly, Na_3_Cit·2H_2_O (0.72g) was dissolved in ethylene glycol (40 ml), named solution A. FeCl_3_·6H_2_O (1.65g) was dissolved in ethylene glycol (30 ml), named solution B. NaAc (3.63g) was dissolved in ethylene glycol (20 ml), named solution C. Then solution B and solution C were added into solution A respectively with stirring, and the mixed solution was in hydrothermal reactor (YK-200, Shanghai Yukang science and Education Equipment Co., Ltd) under 200 ℃ for 10 h. Fe_3_O_4_ nanoparticle was collected and washed for three times with water and ethanol alternately before vacuum drying.

**Determination of CDM loading efficiency**

CMPP (10 mg) obtained by 2.3 was ultrasonically dispersed in THF, and kept stirring until THF completely volatilized. Then shacking washing with THF and separated by the NIB magnet twice while the supernatant was collected, respectively. The absorbance of the supernatant fluid was measured at 203 nm by UV-vis. All measurements were conducted in triplicate. The CDM-loaded rate (LR) was calculated as follows:

$$LR\left( \% \right)=\left( m_{0}-\sum m_{i} \right)/{m_{0}}$$

Where m_0_ was the total mass of CDM, m_i_ was the mass of CDM in supernatant fluid of each shacking washing. Besides, the CDM-THF standard curve was set in **Fig. S1**.


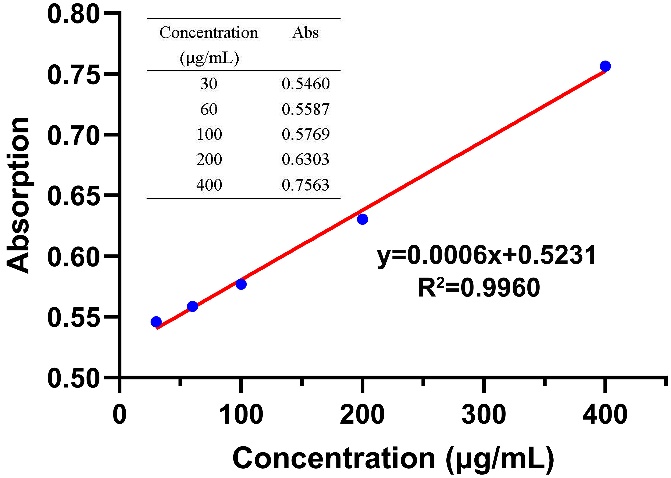


**Figure S1** Standard curve of CDM in THF.


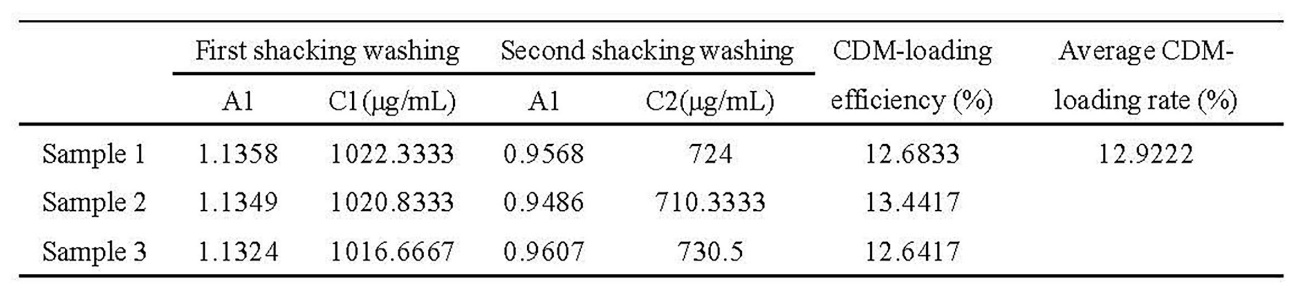


**Table S1** CDM-loading efficiency in MPP (A: absorbance of the remained CDM at 203nm. C: concentration of the remained CDM).


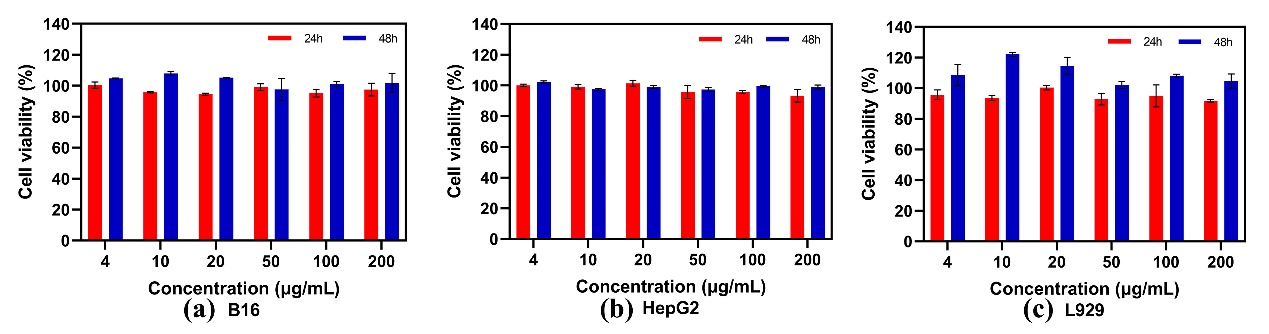


**Figure S2** Biocompatibility of MPP. Cytotoxicity on a) B16-F10, b) HepG2, c) L929.
